# Ultrasound-Assisted Extraction of Humic Substances from Peat: Assessment of Process Efficiency and Products’ Quality

**DOI:** 10.3390/molecules27113413

**Published:** 2022-05-25

**Authors:** Dominik Nieweś, Marta Huculak-Mączka, Magdalena Braun-Giwerska, Kinga Marecka, Aleksandra Tyc, Marcin Biegun, Krystyna Hoffmann, Józef Hoffmann

**Affiliations:** 1 Department of Engineering and Technology of Chemical Processes, Faculty of Chemistry, Wroclaw University of Science and Technology, Wybrzeże Wyspiańskiego 27, 50-370 Wrocław, Poland; magdalena.braun@pwr.edu.pl (M.B.-G.); kinga.marecka@pwr.edu.pl (K.M.); aleksandra.tyc@pwr.edu.pl (A.T.); marcin.biegun@pwr.edu.pl (M.B.); jozef.hoffmann@pwr.edu.pl (J.H.); 2 Department of Micro, Nano and Bioprocess Engineering, Faculty of Chemistry, Wroclaw University of Science and Technology, Wybrzeże Wyspiańskiego 27, 50-370 Wrocław, Poland; krystyna.hoffmann@pwr.edu.pl

**Keywords:** fulvic acids, ultrasound-assisted extraction, UV-Vis spectra, FTIR spectra

## Abstract

Results of efficiency of obtaining humic substances (HSs) from peat in traditional alkaline extraction (TAE) and ultrasound-assisted alkaline extraction (UAAE) are presented. The influence of the duration of the process and ultrasound intensity on the efficiency of extraction of humic acids (HAs) and fulvic acids (FAs) extraction was determined. The composition of the fulvic acid fraction was examined depending on the type of eluent used. Fulvic acids were divided into fractions using columns packed with DAX-8 resin. For this process, 0.1 M NaOH and 0.5 M NH_3_∙H_2_O were used as eluents. For the quality assessment of specific fulvic acids fractions, spectroscopic methods (UV-Vis and FTIR) were used. Ultrasound had a positive effect on HS extraction efficiency, especially in increasing the amount of a desired hydrophobic fraction of fulvic acids (HPO). However, a negative effect of the excessive prolongation and ultrasound intensity (approximately 400 mW∙cm^−2^) on the extraction efficiency of HPO eluted with 0.1 M NaOH solution was observed. Using peat as a raw carbon material for the HS extraction process can be used as an alternative industrial application of peat. UAAE may be considered as an alternative method to TAE, which provides a higher efficiency in HS isolation from peat.

## 1. Introduction

Due to the numerous and valuable properties of humic substances (humic and fulvic acids), preparations produced from alternative organic materials, a rich source of these, such as carboniferous materials with various levels of carbonization, have been widely used in agriculture, chemistry, pharmacy, medicine, and industry in general as well as in other commercial branches. However, it is the humic acid fraction in the fertilizer industry that is the most widely used, and the production process of humic substances from alternative organic materials on an industrial scale is primarily aimed at obtaining a solid humic fraction with sufficiently high efficiency. Due to their lower molecular weight, higher content of functional groups, and therefore greater biological activity than humic acids, fulvic acids are characterized by great application potential in various industries, although they usually appear as a minor component of humic preparations. A comprehensive approach to the efficient process of the production of both valuable fractions is therefore essential. A determining factor influencing the chemical character of humic and fulvic acids as well as the efficiency of their extraction from potential organic sources, aside from the selection of raw materials, is the isolation procedure [1,2].

Generally, humic substances (HSs) are defined as macromolecular compounds whose structures are non-strictly defined. Theories of HS structure describe these substances as molecules with polycyclic, aromatic rings, and aliphatic chains. multiple functional groups that often define the chemical properties of humic substances are linked to the core of the molecule. Humic substances are formed in humification. Process parameters play an important role for the humification, with particular reference to the characteristics of raw material, microorganisms’ presence, temperature, and pH value. The process of humic substances’ formation is biochemical in nature. A description of the HS formation process includes two main theories. According to Waksman’s hypothesis, lignins are particularly decomposed by microorganisms. Biochemical changes of the raw material include a reduction in methacrylic groups, with simultaneous formation of *o*-hydroxyphenols. Carboxyl groups in the side chains are oxidized. As a result of further biochemical processes, humic substances are formed. A second explanation for HSs’ formation is called the polyphenolic theory. This hypothesis assumes HSs’ formation from various raw carbon materials, not only from lignin. Within the polyphenolic theory, two main conceptions are given: Flaig’s and Koronova’s theories [3,4,5,6].

Due to the complicated and long-term process of these materials’ natural formation, it is necessary to take actions aimed at stabilizing and increasing their resources. The carboniferous substances such as peat, lignite, and leonardite are mainly used as source materials for the HS extraction process. River and marine sediments are used as raw materials for this process as well. However, the idea of obtaining humic and fulvic acids from deposits of lignite or peat tends to reconcile economic development with the rational management of non-renewable resources for non-energetic purposes and the global balance of ecosystems [7,8,9,10,11,12].

Alkaline extraction is the basic action undertaken in the process of the production of humic substances from various organic materials. Combinations of physical, chemical, or biological parameters can intensify the extraction of humic acids from the said organic material due to their addition both prior to and following the proper alkaline extraction. These allow for obtaining a similar amount of humic substances from a given mass of raw material in a much shorter time.

Humic substances are mainly divided based on the difference in their solubility at different pH values, and this property is used in the process of their extraction. According to this criterion, humic substances may be divided into humins, which are dissoluble at all pH ranges; humic acids (Has), which are soluble at the alkaline range; and fulvic acids (FAs), which are soluble at all pH range. The hypothesis of humic substances’ structure is mainly based on the polymeric structure of HSs. According to this theory, fulvic acids may be formed by partial oxidation and decomposition of humic acids [6,13,14].

Despite the theories about the structure of humic substances, the determination of strictly described structural models of fulvic acids is still difficult. Qin et al. demonstrated that FAs with a lower molecular mass have greater participation of oxygen functional groups in their structure. Furthermore, fulvic acids have the greatest apparent area and the narrowest micropore distribution among all types of HS fractions [15,16,17,18]. Due to their chemical composition and spatial structure, FAs have a high affinity to metal ions and may be used to bind heavy metal ions [18,19,20,21].

Among other applications of humic substances, including FA fractions, it is important to mention the positive effect of HSs on soil structure, e.g., by improving its sorption capacity. Therefore, many studies on the applications of humic substances focused on agriculture, soil science, and the possibility of using HSs as a component for commercial fertilizers’ production. Research is also being carried out on FA’s application as a component for animal feed [22,23,24,25,26].

Due to the lack of a clear and unambiguous definition for the group composition of fulvic acids, these humic substances are mainly fractionated based on their hydrophilic or hydrophobic character. That parameter can also determine different properties of various FA fractions. According to that methodology, fractionation of fulvic acids is carried out based on the difference of affinity to the hydrophobic or hydrophilic ion-exchange resins. Resins that adsorb the hydrophobic fraction of FAs (e.g., XAD-8, DAX-8, XAD-7HP) are mainly used for this purpose. Those types of resins are defined as macro-porous sorbents based on styrene or acrylic esters, which can bind hydrophobic substances through weak van der Waals or hydrogen bonds. Fractionation on DAX resins is carried out based on the differences in the polarity [27,28,29]. A fraction that is adsorbed on the DAX resin and then desorbed in alkali solution is referred to as a hydrophobic fraction, or HPO, while a hydrophilic fraction (HPI) passes through the resin into the effluent. Different fractions are characterized by different functional groups. Aromatic phenol groups are more commonly associated with the HPO fraction. The HPI fraction contains more aliphatic and carboxyl carbons and nitrogenous compounds, such as low-molecular-weight fulvic acids, as well as carbohydrates, proteins, and amino acids, which are an undesirable component of the fraction in the context of their potential use [30,31].

For the intensification of HSs extraction, ultrasound-assisted alkaline extraction (UAAE) and microwave-assisted alkaline extraction (MAAE) are used. Raposo et al. [32] proved the significant effect of the UAAE process on increasing the repeatable efficiency of humic substances obtained from organic raw materials compared to traditional alkaline extraction (TAE) using a bath shaker. An important part is also the preparation of raw material with acidic treatment. This method is recommended by the International Humic Substances Society (IHSS). The use of modern techniques and pre-treatment in the extraction of humic acids from potential organic materials improves and reduces the cost of this process and shortens its duration [33,34]. Ultrasound-assisted extraction is widely applied for the isolation of many natural products from organic raw materials. UAE is a green alternative for natural bioproducts’ extraction for food, pharmaceutical, and cosmetic applications. Using ultrasound-assisted extraction allows for green concentrates to be obtained. Application of UAE methods is a compromise between the efficiency and the cost of the extraction procedure at an industrial scale [35]. Tiwari noted that the application of UAE allows for replacement of the traditional extractant with eco-friendly alternatives, while ensuring the same efficiency for the extraction process. Among the main advantages of the UAE process is the possibility of enhancing the aqueous extraction without aggressive solvent and enhancing the extraction efficiency for heat-sensitive compounds by using the milder conditions of the extraction process [36]. Low-frequency ultrasound cavitation is applied as a non-thermal procedure for extraction intensification. That process is used for metabolite extraction (e.g., polyphenols and flavonoids) from food production wastes [37].

Using the modified method of humic substance extraction is especially interesting for improving the efficiency of HS extraction from raw carbon materials such as peat, lignite, or leonardite. The aforementioned research on ultrasound-assisted extraction was focused on HS extraction from marine sediments. The UAE method may be also applicable for HS extraction from other sources, especially from raw carbon materials.

For the assessment of the chemical structure of humic substances, spectroscopic methods (e.g., FTIR and UV-Vis) are used. Ultraviolet-visible spectroscopy methods are especially useful for determining the interactions between fulvic acids and metal ions. Due to the development of UV-Vis methods for fulvic acids, it is possible to describe the interactions between FAs and heavy metals or micronutrients [38,39,40,41]. UV-Vis may also be an interesting alternative to traditional methods of HS identification, especially for describing the character of FA fractions, which are in a raw solution after precipitation of humic acids. Fourier transform infrared spectroscopy (FTIR) can be used as a complementary instrumental method for rapid quality control of HSs with minimum sample preparation. It is sensitive to the main functional groups of HSs. The advantages of FTIR spectroscopy versus fluorescence spectroscopy are: less dependent on accompanying factors such as light scattering and lack of signal quenching [42].

## 2. Results and Discussion

The efficiencies of the HAs and raw extracts of the FA fractions were calculated for dry-ash-free conditions (daf). The results of the extraction efficiency for humic acids (HA^daf^) and raw extracts of fulvic (FA^daf^) acids are presented in Table 1. They were referenced to the mass of the raw material. The fulvic acid extract that was isolated after precipitation of the humic acids was defined as the raw extract of the fulvic acids. That mixture, except for the essential fraction of FAs (HPO), also consisted of other types of organic compounds that may have been extracted from the peat.

The application of the UAAE process for the extraction of humic substances increased the efficiency of the HAs and the raw extract of FAs obtained from peat. The highest extraction efficiency of HAs, equal to 56.70 mass%, was obtained for UAAE, which was carried out in 90 min with an ultrasound intensity equal to 300 mW∙cm^−2^. The combination of increased process time and ultrasound intensity (135 min and 400 W∙cm^−2^) caused the decrease in the HA extraction efficiency and an increase in the FA content. According to the polymeric hypothesis of the structure of humic substances, the increase in the process time and intensification of the ultrasound intensity caused the degradation of the structure of humic acids and led to their disintegration into the fraction of fulvic acids. HA degradation is probably also caused by the intensification of stirring under the aerobic conditions that resulted from a higher ultrasound intensity. Aerobic degradation of humic acids was described by Anđelković et al. [43].

**Figure 1:**
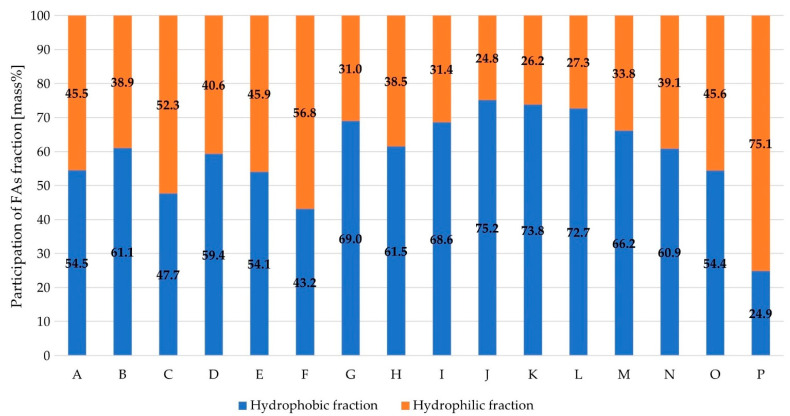
Participation of hydrophilic and hydrophobic fractions from the separation of fulvic acids, obtained from peat, in the TAE and UAAE processes under various initial parameters.

The results of the participation of various fractions of FAs, depending on the type of extraction and initial conditions of the process, are presented in Figure 1. The efficiencies of fulvic acid extraction in the TAE and UAAE processes were compared. In total, 16 various samples (A-P) of FA fractions were analyzed. They were marked according to Table 2. Detailed conditions of the extraction and separation processes for FA hydrophilic and hydrophobic fractions are presented in Section 3 (Materials and Methods).

For ultrasound-assisted alkaline extraction, compared to the traditional extraction process, a greater participation of the hydrophobic fulvic acid fraction (HPO) was obtained. Only the last point (P) was an exception. The longer duration of the TAE and UAAE processes caused the higher amount of the hydrophilic fraction in the total FAs that were extracted from the raw carbon materials. The UAAE process, with had both the longest duration and the highest ultrasound intensity, caused a significant reduction in the HPO fraction, which was eluted by a 0.1 M NaOH solution, in the total amount of FAs (point P). Increasing both process parameters (time and ultrasound intensity) probably led to changes in the structure of fulvic acid fractions with the intensification of the hydrophilic fraction (HPI) obtained. Mainly non-humic substances (i.e., polysaccharides, amino sugars, amino acids, proteins, fatty acids, carbohydrates, lipids, etc.) belong to this fraction, which, from the point of view of the extraction process, artificially increases the amount of proper fulvic acids. This observation is connected with the fact that stronger conditions of extraction of humic substances also cause the extraction of other substances present in the raw material.

**Figure 2:**
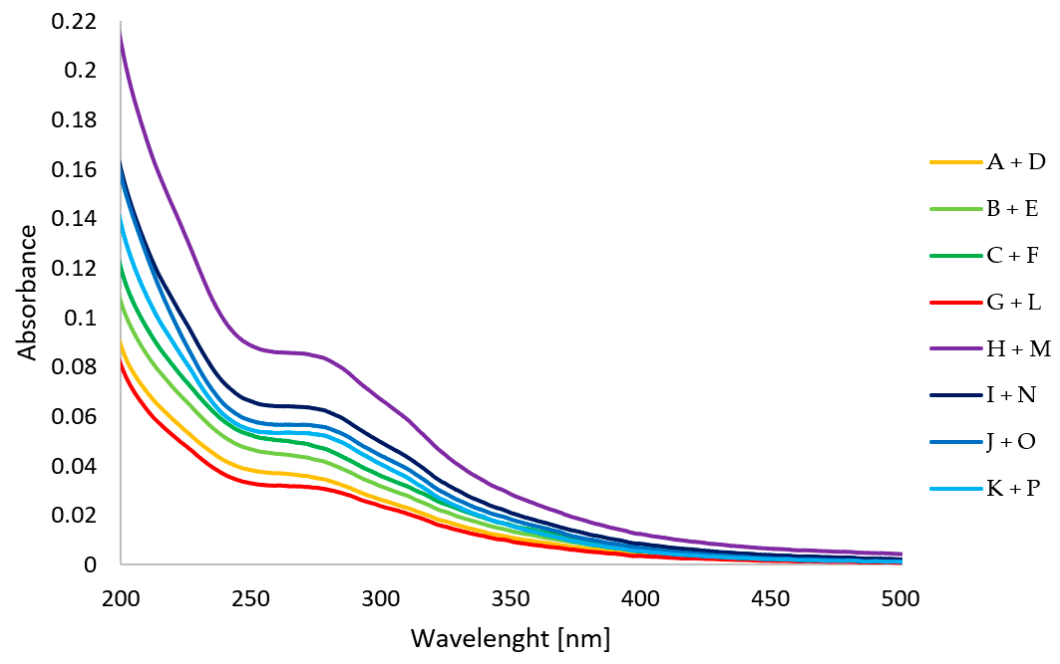
UV-Vis spectra of the fulvic acid fractions before the fractionation step using the DAX-8-packed column. Each sample was described as the sum of HPO and HPI fractions, using the symbols presented in Table 2.

The first step of qualitative evaluation of fulvic acids, including the HPO and HPI fractions, was carried out using UV-Vis spectroscopy. The detailed procedure for quality assessment is presented in the Materials and Methods section. The UV-Vis spectra of FAs before fractionation using a column with DAX-8 are presented in Figure 2. Then, the evaluation of the HPI and HPO fractions was performed. For a clear presentation of the UV-Vis spectra results, each sample was determined, according to Table 2. For the results presented in Figure 2, each sample was described as the sum of HPO and HPI fractions.

Depending on the extraction method, the differences in the course of the curves presented in Figure 2 were observed. For the TAE process, the UV-Vis spectra had a more monotonic character. The differences in UV-Vis spectra, depending on the various extraction methods, demonstrated transformations of the structure of the fulvic acids. That was especially observed for samples extracted in the UAAE process using the highest ultrasound intensity. In the ultraviolet range, local absorption peaks were observed. This indicated the presence of chromophores. Between UV-Vis spectra, hyperchromic and hypochromic effects were defined. For some spectra, which are presented in Figure 2, the maximum absorbance peaks for the wavelength equal to 280 nm were observed. That is characteristic of the C=O chromophore with valence electron excitation. On the basis of UV-Vis spectra for a mixture of HPO and HPI fractions, it can be concluded that fulvic acids contain oxygen atoms in the structures of their rings [6].

**Figure 3:**
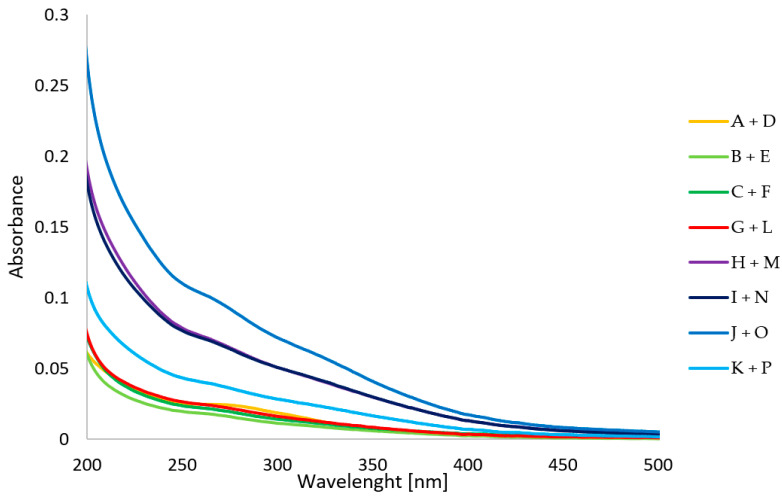
UV-Vis spectra of HPI fractions after fractionation using a DAX-8-packed column. The samples are described using symbols from Table 2.

After fulvic acid fractionation, using a column packed with DAX-8 hydrophobic resin, the HPI fraction was separated, and the HPO fraction was absorbed. In Figure 3, UV-Vis spectra are presented for hydrophilic fractions of fulvic acids extracted from peat using various extraction types and process parameters. For the description of the samples presented in Figure 3, symbols from Table 2 are used. As a result of not having to use the eluent for the HPI fraction, separating each sample of HPI fraction was described as the sum of samples that were obtained with the same type of extraction and process parameters.

In Figure 3, a lack of local absorbance peaks at 280 nm was observed, indicating the lack of chromophores, which are characteristic of the hydrophobic fractions of the fulvic acids. Based on the UV-Vis spectra presented in Figure 3, it can be concluded that the samples of the HPI fractions had a minimal content of essential fulvic acids, the presence of which may be characterized by an absorbance peak in the ultraviolet light.

**Figure 4:**
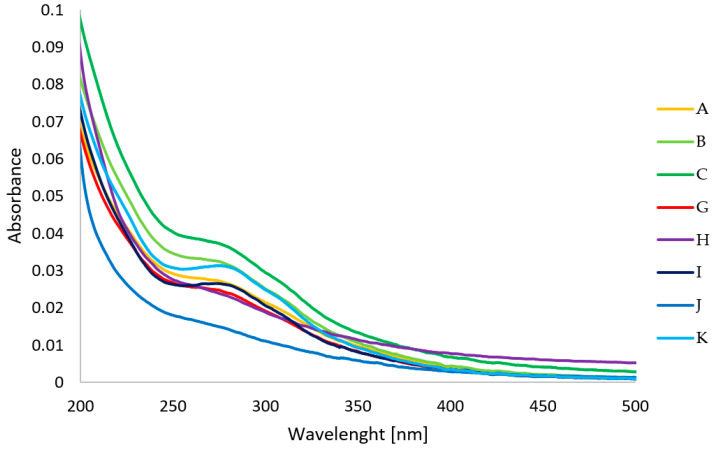
UV-Vis spectra of HPO fractions after fractionation using a DAX-8-packed column and eluted with 0.5 M NH_3_∙H_2_O. Samples are described using symbols from Table 2.

**Figure 5:**
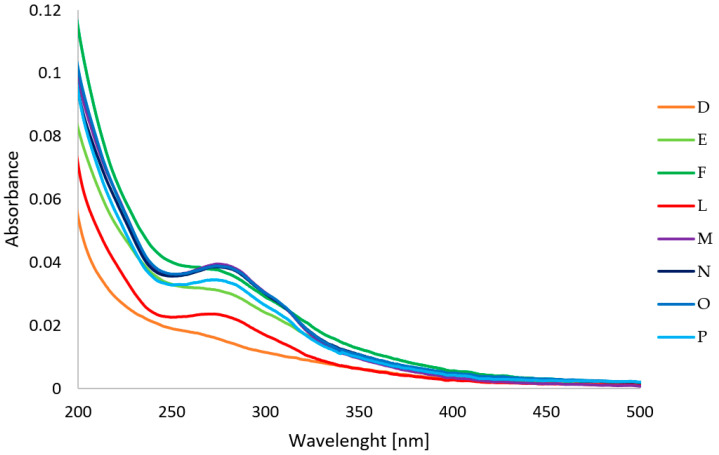
UV-Vis spectra of HPO fractions after fractionation using DAX-8-packed column and elution with 0.1 M NaOH. Samples are described using symbols from Table 2.

UV-Vis spectra for the HPO fractions are presented in Figure 4 and Figure 5. For the elution of the essential fraction from the column packed with DAX-8 resin, two types of eluents (0.1 M NaOH and 0.5 M NH_3_∙H_2_O) were used. The presented results refer to both extraction processes (TAE and UAAE). The samples are marked according to Table 2.

UV-Vis spectra for HPO fractions that were extracted by the TAE and UAAE processes were similar. Hyperchromic and hypochromic effects between the obtained spectra were observed. In Figure 4 and Figure 5, the local peaks at a wavelength equal to 280 nm were observed. This is an indication of the absorption of the essential hydrophobic fraction of fulvic acids by DAX-8 resin. In the next step, the HPO fraction was eluted from the column using two types of eluents. As a result of the successful separation of FAs, a hydrophobic fraction, which included the essential part of fulvic acids, was obtained. The HPO fraction had optical properties, in contrast to the HPI fraction, which mainly included other types of extracted organic components, without essential fulvic acids. Using 0.1 M NaOH and 0.5 M NH_3_∙H_2_O as eluents was effective in removing HPO from the column packed with hydrophobic DAX-8 resin.

The research was based on the extraction process for humic substances recommended by the International Humic Substances Society (IHSS), with the author’s modification, which involved changing the concentration of the solutions and avoiding the use of HF. An important part of the applied method was the preparation of the peat by acidic pre-extraction. The modified procedure of HSs isolation in combination with the UAAE process increased the efficiency of HSs extraction, rapidly extracting the desired hydrophobic fraction of fulvic acids from raw carbon material, and resulted in high reproducibility, while the consumption of reagents was reduced and energy requirements were lowered. For the quantitative assessment of HSs, the main fractions that were obtained after precipitation of HAs, the gravimetric method was applied [44]. Using the modified method, based on the procedure described in the ISO 19822:2018 standard, allowed fast assessment of the content of the two main fractions in the extract obtained.

For the quantitative assessment of fractions after the distribution of FAs into two groups (HPO and HPI), the titrimetric method, based on the procedure described in ISO 5073:2013, was used [45]. Determination of FAs after fractionation by titration is more sensitive compared to the gravimetric method. The combination of the two types of quantitative analysis for HSs allowed for a complex assessment for all fractions that were obtained in the presented study.

UV-Vis spectroscopy was used for the initial quality assessment of the HPO and HPI fractions. The application of a simple method for the determination of the hydrophobic fraction that consists of an essential type of FA allowed for the fast evaluation of the effectiveness of fractionation. The experimental determination of the wavelength was based on the identification of the peak, which was specified for a hydrophobic fraction of FAs, using a column packed with DAX-8 resin after separation.

**Figure 6:**
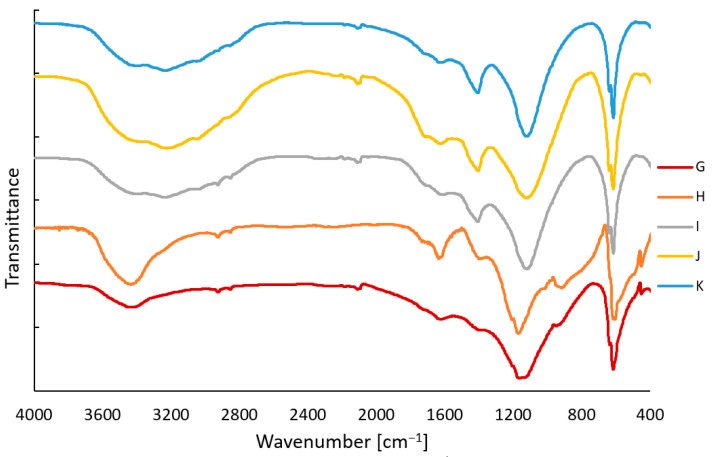
FTIR spectra of HPO fractions after fractionation using DAX-8-packed column and elution with 0.5 M NH_3_∙H_2_O. Samples are described using symbols from Table 2.

**Figure 7:**
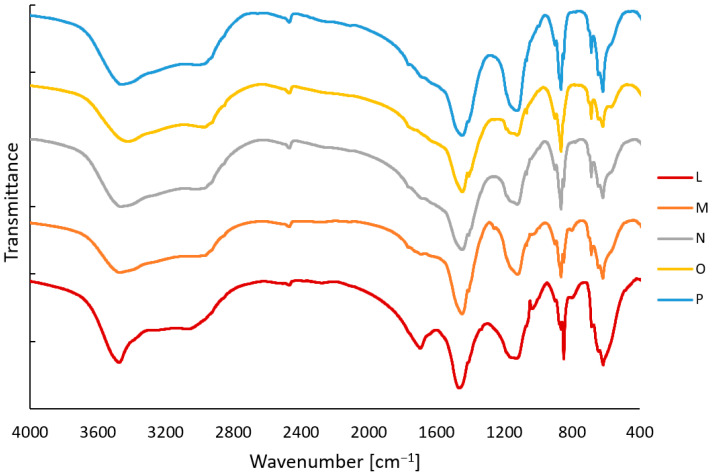
FTIR spectra of HPO fractions after fractionation using DAX-8-packed column and elution with 0.1 M NaOH. Samples are described using symbols from Table 2.

A comparison of UV-Vis with another spectroscopic method for the quality assessment of humic substances (FTIR) was carried out for HPO samples obtained through the UAAE process. The influence of ultrasound on the presence of characteristic functional groups in the essential part of FAs was assessed on that basis. The results of the FTIR analysis are grouped in view of the type of eluent. Spectra for HPO samples eluted with 0.5 M NH_3_∙H_2_O are presented in Figure 6, and samples eluted with 0.1 M NaOH are demonstrated in Figure 7. Infrared bands were interpreted according to descriptions by Stevenson [6], Raposo et al. [32], Romaris-Hortas et al. [34], and Santos et al. [46]. Infrared spectra were slightly different for HPO samples eluted with different solutions. However, all spectra were characterized by a broad band in the region 2900–3600 cm^−1^, which corresponds to the stretching of the O–H of phenol, alcohol, carboxylic acid, or water. Weak peaks around 2950 and 2850 cm^−1^, characteristic of HPO eluted with 0.5 M NH_3_∙H_2_O, were reported to be caused by stretching vibrations of aliphatic C–H bonds in the CH_3_ and CH_2_ groups. The main differences between the samples could be observed in the region from 800 to 1800 cm^−1^. The absorption band at about 1700 cm^−1^ may be attributed to the vibrations of C=O stretches of ketones and carboxylic acids. It is particularly evident for HPO samples that were obtained by low-intensity ultrasound. The band at 1400–1500 cm^−1^, which was particularly evident for HPO samples eluted with 0.1 M NaOH, was probably caused by the C–H deformation of the aliphatic structures. Another band could be distinguished in the region 1000–1260 cm^−1^ and could be attributed to the C–O stretching of esters, phenols, alcohols, and polysaccharides. The peak at around 800–850 cm^−1^ was attributed to the C–H of the substituted aromatic groups. That signal was characteristic of the samples whose spectra are presented in Figure 7. A strong peak around 550 cm^−1^ arose from the mineral contamination. This was possible because of the lack of the step of HPO purification using the HF solution. On the other hand, according to Chang et al. [47], that signal may also be attributed to the S–S vibrations.

Presented results show that the UAAE process may be applied as an alternative to the TAE process. However, a negative effect of the excessive prolongation and ultrasound intensity (approximately 400 mW∙cm^−2^) on the extraction efficiency of HPO eluted with 0.1 M NaOH solution was observed. Therefore, for effective isolation of high-quality HS fraction, an appropriate choice of parameters for the UAAE process (especially ultrasound intensity) is important. Moreover, the results show the influence of the type and concentration of the eluent on the efficiency of isolation of the HPO fraction.

## 3. Materials and Methods

### 3.1. Materials

NaOH, NH_3_∙H_2_O (25 mass%), HCl, and H_2_SO_4_ were purchased from POCH (Avantor Performance Materials Poland S.A., Gliwice, Poland). For solutions’ preparation, deionized water (<1.0 $\mu S\cdot {\mathrm{cm}}^{-1})$ was used. Humic substances were extracted from peat. The raw carbon material came from the marshes of the Wisła estuary (Żuławy district). The peat was sampled from six different drillings, which were carried out at a depth from 0.5 to 1.5 m. Observation of its decomposition allowed us to define that material as a pseudo-fibrous peat (H5−H7 on the von Post scale). Before the HS extraction procedure, the peat was air-dried until the moisture content was approximately 65 mass%.

Before the main HS extraction process, raw carbon material was prepared by acidic pre-extraction. For this step, 0.1 M and 1 M solutions of hydrochloric acid were used. For pH stabilization, 1 M NaOH was used. The alkaline substance, which was used to extract humic substances from peat, was 0.5 M NaOH. Humic acids were precipitated from the extract using 1 M H_2_SO_4_. Fulvic acid fractionation in hydrophobic (HPO) and hydrophilic (HPI) fractions was carried out using a column packed with Supelite^TM^ DAX-8 hydrophobic resin, which was acquired from Sigma-Aldrich (St. Louis, MO, USA). Before the FA separation process, the resin was prepared by soaking in HPLC-grade methanol, which was purchased from Sigma-Aldrich (St. Louis, MO, USA).

For the elution of the HPO fraction, two types of eluents (0.1 M NaOH and 0.5 M NH_3_∙H_2_O) were used. The organic carbon content was determined using: 0.068 M K_2_Cr_2_O_7_, H_2_SO_4_ (96 mass%), 0.02 M KMnO_4_ (prepared using the analytical portion of the TitraFix^TM^ sample), and 0.2 M Fe(NH_4_)_2_(SO_4_)_2_·6 H_2_O (Mohr’s salt). Ferroin sulfate solution was used as the indicator. For the spectroscopic research step, the pH range was regulated by 0.01 M NaOH and 0.025 M H_2_SO_4_ solutions. All reagents for the determination of the organic carbon content were purchased from POCH (Avantor Performance Materials Poland S.A., Gliwice, Poland).

Raw carbon material pre-extraction and the extraction of HSs in the TAE process were carried out using a water bath shaker Elpan type 357.

Gravimetric determination of HSs was performed using a laboratory POL-EKO SL115 drying oven (POL-EKO Aparatura, Wodzisław Śląski, Poland) and Czylok FCF12 SHM muffle furnace (Czylok Company, Jastrzębie-Zdrój, Poland). Fulvic acid solutions, before determination using the gravimetric method, were concentrated using BUCHI ROTAVAPOR R-100 vacuum rotary evaporation (BÜCHI Labortechnik AG, Flawil, Switzerland). The pH value was measured by the laboratory pH controller Orion 2-Star (Thermo Scientific, Waltham, MA, USA). Spectroscopic measurements were made using a V-670 UV-Vis-NIR spectrophotometer (Jasco, Kraków, Poland) and a Nicolet 6700 FTIR spectrophotometer (Thermo Scientific).

**Scheme 1:**
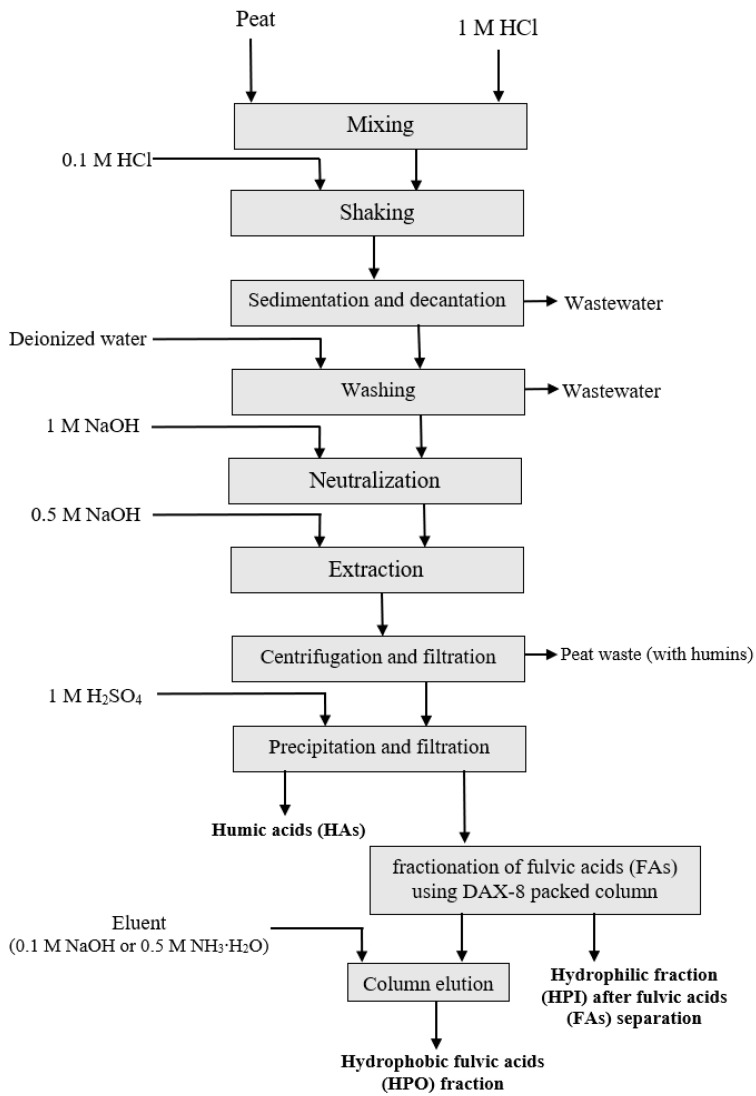
Process of extraction and fractionation of humic substances obtained from peat as a raw carbon material.

The extraction process, which was focused on obtaining HSs from peat, is described as Scheme 1. Its 3 main steps were:

- Acidic pre-extraction of raw carbon material;
- Extraction of HSs from peat (according to the TAE or UAAE processes);
- Separation of HSs into 3 fractions: humic acids (HAs), hydrophobic (HPO), and hydrophilic (HPI) fulvic acids.

For each of the extraction processes, 25 g of peat was used. The extractant-to-raw carbon material (E/CRM) mass ratio was 15:1.

### 3.2. Raw Carbon Material Preparation

The first step of the laboratory-scale extraction process of humic substances was the preparation of raw carbon material. The presented method was recommended by IHSS and allows for the decalcification of raw carbon material. The acidic preparation of peat causes the bond to break between humic substances and calcium compounds. That may provide for the increase in the efficiency of extraction of humic substances from peat. A total of 80 g of peat was mixed with 80 mL of 1 M HCl in a polypropylene bottle until the pH value equaled approximately 2. In the next step, 720 mL of 0.1 M HCl was added to adjust the final mass ratio of the acid solution to the raw carbon material equaled 10:1. The peat with the HCl solution was mixed using a bath shaker at room temperature. After 1 h, the pre-extracted peat was separated and neutralized by deionized water washing. At the final step of peat pH correction, a small amount of 1 M NaOH was added, and after that, the raw carbon material was air-dried for 12 h.

### 3.3. Humic Substances Extraction

Humic substances were extracted from peat using traditional alkaline extraction (TAE) and ultrasound-assisted alkaline extraction (UAAE) processes. Each process was carried out at three various times: 45, 90, and 135 min. Table 2 presents a combination of process parameters for the extraction and fractionation process of HSs. For the extraction processes, i.a., extreme points with minimal or maximal values of the process parameters were chosen. That allowed us to determine the range of values of the process parameters for TAE and UAAE, providing the high level of humic acids and the hydrophobic fraction of the concentrations of fulvic acids in the extract. On the basis of the extremal values of experimental points, it was possible to create an experimental matrix and use statistical methods for describing the dependencies between the values of process parameters and the efficiency of HSs extraction.

### 3.4. Traditional Alkaline Extraction (TAE) Process

A total of 25 g of pre-extracted raw carbon material was mixed with 375 cm^3^ of 0.5 M NaOH in a polypropylene bottle. The samples were shaken at room temperature according to the process parameters, which are described in Table 2. In the next step, the extract of HSs was isolated from the post-extraction waste by centrifugation at 3000 rpm for 15 min and vacuum filtration using qualitative filters. Subsequently, the volume of the extract was determined, and the humic acids were precipitated using 1 M H_2_SO_4_ by reducing the pH value to 2. For complete precipitation of HAs, the acidified extract of HSs was stored at 5 °C for 12 h. A gel of humic acids was separated from the solution of fulvic acids by filtration using quantitative filters. After the separation process, HAs with the filter were inserted into a porcelain crucible. The fulvic acids solution was concentrated using a vacuum evaporator and placed in a porcelain crucible.

### 3.5. Ultrasound-Assisted Alkaline Extraction (UAAE) Process

UAE is defined as a non-destructive method that reduces process time and provides a highly efficient extraction process compared with traditional extraction, where mass transfer is intensified by mechanical stirring. A sample of 25 g of pre-extracted raw carbon material was mixed with 375 cm^3^ of 0.5 M NaOH and placed in the 500 cm^3^ flask. The HSs were extracted in the UAAE process using an ultrasound bath with the ultrasonic generator EMMI 40HC (EMAG, Juszczyn, Poland). The process for all samples was carried out at room temperature according to the process parameters described in Table 2. The ultrasound frequency was equal to 45 kHz for all samples. After the UAAE process, the extract of HSs was separated from the post-extraction waste by centrifugation at 3000 rpm for 15 min. For the precipitation of the HAs fraction, the pH of the extract was reduced to 2 using 1 M H_2_SO_4_. The acidified extract was stored at 5 °C for 12 h. The next steps, including separation of the HA and FA fractions and concentration of fulvic acids, were the same as for the procedure presented in Section 3.4.

### 3.6. Fulvic Acids’ Fractionation

The hydrophobic and hydrophilic fractions of fulvic acids were obtained by separation using a column packed with Supelite^TM^ DAX-8 hydrophobic resin (Sigma-Aldrich, St. Louis, MO, USA). HPO was an essential fraction of FAs, and the HPI fraction consisted mainly of other organic compounds, which were extracted from raw carbon material. Before the fractionation process, the DAX-8 resin was prepared by soaking and mixing with HPLC methanol and deionized water. The hydrophobic resin was then washed using deionized water to remove residual methanol. The column was packed with 0.15 cm^3^ of the prepared DAX-8 resin for each of the 100 cm^3^ fulvic acid solutions, which was passed through the column. A total of 500 cm^3^ of FA solution for each sample was fractionated using a DAX-8-packed column. The flow rate of the FAs was corresponded to the volume of the column bed. The volumetric ratio of FA solutions, which were fractionated by column per hour, to the DAX-8 resin was 15:1. Parts of fulvic acids that were not absorbed in the packed bed should not include essential fulvic acids. The hydrophobic fraction (HPO), representing an essential part of FAs, was absorbed in DAX-8 resin and eluted using 0.1 M NaOH or 0.5 M NH_3_∙H_2_O.

### 3.7. Quantitative Assessment of Humic Substances

For fast assessment of the participation of the FA and HA fractions in the extract, a modified gravimetric method based on the ISO 19822:2018 standard was used [41]. Porcelain crucibles containing HAs and quantitative filter or concentrated FAs were dried at 105 °C for 24 h. In the next step, the samples were incinerated at 615 °C for 5 h. The efficiencies of the extraction of HSs using TAE or UAAE processes were determined by the amount of HAs or FAs that was extracted from raw carbon material and compared to the dry-ash-free basis (DAF) of peat. To calculate the efficiency of obtaining humic acids (*HA^daf^*), in mass%, Equation (1) was used.

(1) $$HA^{daf}=\frac{m_{1}-m_{2}-m_{3}}{m_{4}\cdot \left(100-W^{a}-A^{a}\right)}\cdot \frac{V_{0}}{V_{1}}\cdot 10\;000$$

FA extraction efficiency, in relation to the dry-ash-free raw carbon material (*FA^daf^*), was defined in mass% and calculated using Equation (2):

(2) $$FA^{daf}=\frac{m_{1}-m_{2}}{m_{4}\cdot \left(100-W^{a}-A^{a}\right)}\cdot \frac{V_{P}}{V_{2}}\cdot 10\;000$$

where *m*_1_ is the mass of dry humic or fulvic acids [g]; *m*_2_ is the mass of ash after the incineration of HAs or FAs [g]; *m*_3_ is the mass of the quantitate filter [g]; *m*_4_ is the mass of peat, which was used as a raw carbon material in the extraction processes [g]; *W^a^* is the moisture content in an analytical sample of peat [mass%]; *A^a^* is the ash content in the analytical sample of raw carbon material [mass%]; *V*_0_ is the total volume of the HS extract [mL]; *V*_1_ is the extract volume, which was used for the HAs precipitation [mL]; *V*_2_ is the volume of fulvic acids solutions, which was used for concentration and gravimetric analysis [mL]; and *V_p_* is the total volume of FA solution [mL].

For the determination of the organic carbon content (C_org_.) in the HPO and HPI fractions of the FA phase, a modified method based on ISO 5073:2013 standard was used [44]. The analytical method was based on C_org_. oxidation to CO_2_ using 0.34 M K_2_Cr_2_O_7_ in the presence of H_2_SO_4_ and heating of the mixture, according to the chemical reaction:

$$3C_{\mathrm{org}.}+2K_{2}{\mathrm{Cr}}_{2}O_{7}+8H_{2}{\mathrm{SO}}_{4}\to 3{\mathrm{CO}}_{2}+2{\mathrm{Cr}}_{2}{\left({\mathrm{SO}}_{4}\right)}_{3}+2K_{2}{\mathrm{SO}}_{4}+8H_{2}O$$

The samples were quantitively transferred to 100 mL volumetric flasks and mixed with deionized water. During the analysis of C_org_., excess oxidizing agent was titrated using Mohr’s salt solution in the presence of ferroin sulfate as an indicator, according to the reaction below:

$$K_{2}{\mathrm{Cr}}_{2}O_{7}+7H_{2}{\mathrm{SO}}_{4}+6{\mathrm{FeSO}}_{4}\to {\mathrm{Cr}}_{2}{\left({\mathrm{SO}}_{4}\right)}_{3}+K_{2}{\mathrm{SO}}_{4}+7H_{2}O$$

The color changed from orange to red, marking the end of the titration. Blanks were prepared using deionized water instead of HSs. Organic carbon content, in mass%, was calculated according to Formula (3):

(3) $${\%C}_{\mathrm{org}}=\frac{\left(a-b\right)\cdot n\cdot 0.003}{V}\cdot r\cdot 1000$$

where *a* is the volume of 0.2 M Fe(NH_4_)_2_(SO_4_)_2_·6H_2_O solution, which was used for blank titration [mL]; *b* is the volume of 0.2 M Fe(NH_4_)_2_(SO_4_)_2_·6H_2_O solution, which was used for organic sample titration [mL]; *r* is the sample dilution factor, *n* is the molar concentration of Mohr’s salt solution [moL/L]; and 0.003 and 1000 are correction coefficients. The participations of the HPO and HPI fractions in the total amount of FAs were calculated by the ratio of C_org_. content in a given fraction and the organic carbon content in FAs, which was a mixture of hydrophobic and hydrophilic fractions.

### 3.8. Quality Evaluation of Fulvic Acid Fractions

The UV-Vis spectroscopy procedure was implemented for FAs, HPI, and HPO. A total of 1 mL of sample was entered into a 200 mL volumetric flask and mixed with deionized water. The pH of the diluted sample was corrected by adding 0.01 M NaOH and 0.025 M H_2_SO_4_ solutions. For the prepared samples, the absorbance was measured. The spectrophotometer correction was performed using deionized water. For FTIR analysis, HPO samples were dried at 65 °C for 72 h. A total of 1.5 mg of each dried sample was mixed with 300 mg of KBr [48], compressed, and analyzed between 4000 and 400 cm^−1^.

## 4. Conclusions

The aim of the presented work was to compare ultrasound-assisted alkaline extraction (UAAE) with traditional alkaline extraction (TAE) for the process of isolating humic substances from peat. Using gravimetric methods, analytic determination of organic carbon (C_org_.) content was performed for the quantitative assessment of the humic substances. The UV-Vis technique was used for the identification of the hydrophobic fractions of fulvic acids (HPO). The functional groups were determined by the FTIR method. The application of the UAAE process, compared with TAE, caused a higher efficiency in obtaining HSs. However, the maximization of the intensity of ultrasound and the duration of the process led to decreased efficiency in extracting humic acids.

The raw extract of fulvic acids was divided into fractions using a column packed with DAX-8 resin. For the fractionation process, the effectiveness of two types of eluents (NaOH and NH_3_∙H_2_O) was proved. For UV-Vis spectra of HPO, peaks of absorbance were observed. FTIR spectra revealed vibrations of groups characteristic of the fulvic acid fraction. Signals from aliphatic structures dominated. C=O stretches were observed only for HPO samples, which were extracted in a low-intensity UAAE process. The lack of a peak at about 1700 cm^−1^ for other samples was probably caused by blockage of C=O vibrations by aliphatic chains, which were dominant in the analyzed HPO samples. Spectroscopic methods may be used for the preliminary identification of the hydrophobic fraction of fulvic acids. An important part of the presented work was to determine the extremal conditions for the UAAE process. The combination of an ultrasound intensity of about 400 mW∙cm^−2^ and a process duration greater than 120 min caused a significant decrease in product quality due to the minimization of humic acids and HPO contents, which can be eluted by 0.1 M NaOH.

The application of the UAAE method for the extraction of HSs from raw carbon materials may be an interesting green alternative to traditional extraction. On the basis of the presented results, it is possible to describe the range of process conditions for UAAE that were applicable for the extraction of HSs from peat that allowed us to obtain high-quality humic substance extracts in an eco-friendly process. The proposed UAAE method may allow for an increase in the efficiency of HS isolation from raw materials. Industrial implementation of alkaline extraction assisted by low-intensity ultrasound can increase the cost-effectiveness of obtaining humic substances for various purposes, e.g., for fertilization. Moreover, it allows for the implementation lower-toxicity solvents while maintaining appropriate efficiency of isolation process.

## Figures and Tables

**Table 1:** Efficiency of humic and fulvic acid extraction in TAE and UAAE processes.

Extraction Method	Ultrasound Intensity [mW∙cm^−2^]	Time [min]	HA^daf^ [mass%]	FA^daf^ (Raw Extract) [mass%]
TAE	N/A	45	22.60	21.95
		90	26.50	26.55
		135	28.95	29.80
UAAE	200	45	34.20	34.15
	200	135	56.09	36.10
	300	90	56.70	37.40
	400	45	54.51	38.93
	400	135	52.92	40.55

**Table 2:** Combination of process parameters for experimental points (A–L) of humic substance extraction from peat.

Sample Designation	Extraction Type	Eluent Type for FAs Fractionation ^1^	Ultrasound Intensity [mW∙cm^−2^]	Time [min]
A	TAE	NH_3_∙H_2_O	N/A	45
B				90
C				135
D		NaOH	N/A	45
E				90
F				135
G	UAAE	NH_3_∙H_2_O	200	45
H			200	135
I			300	90
J			400	45
K			400	135
L		NaOH	200	45
M			200	135
N			300	90
O			400	45
P			400	135

^1^ Eluent type is an additional parameter for UV-Vis spectra assessment of hydrophobic fulvic acid fraction (HPO).

## Data Availability

The data presented in this study are available on request from the corresponding authors.

## Sample Availability

Samples of the compounds are not available from the authors.

## Abbreviations

FAs	fulvic acids
FTIR	Fourier-transform infrared spectroscopy
HPI	hydrophilic fraction of fulvic acids
HPO	hydrophobic fraction of fulvic acids
HSs	humic acids
IHSS	International Humic Substances Society
TAE	traditional alkaline extraction
UAAE	ultrasound-assisted alkaline extraction
UV-Vis	ultraviolet-visible spectroscopy
